# The British Red Cross Society and the Balkan War

**Published:** 1912-11-09

**Authors:** 


### THE BRITISH RED CROSS SOCIETY AND THE BALKAN WAR.

### The Staffing, Pay, and Equipment of the Units.

The advice recently given in the pages of The Hospital to the British Red Cross Society that it should perfect its organisation for general Red Cross work has received ample confirmation in connection with the outbreak of hostilities, that has been the chief interest of late, between the Balkan States and Turkey. Although this country is in no way involved in the conflict and is very properly preserving a strict neutrality in the matter, the nation's sense of humanity could not be indifferent to the fact that a vast amount of suffering was bound to accompany the struggle and that something should be done on our part to help in its alleviation. The only channel through which such assistance could be given was our British Red Cross Society, which has taken action, thus correctly interpreting the public feeling and indicating that it was ready to undertake the duty that belongs to it. So far we must congratulate the Society on the very satisfactory manner in which the work it has taken in hand has been accomplished. It has been carried through expeditiously and effectively, and everything has been done to make the Red Cross units sent out a credit to this country and a real assistance to those who may need their services.

An occasion such as this should be a very valuable experience to the British Red Cross Society, as it not only illustrates in a way nothing else can the advantages of being properly organised to cope with sudden and unlooked for contingencies, but it also brings out any weak points and deficiencies that may exist in the system laid down for rendering aid. It should also emphasise what is not always put into practice, and that is that previously well-arranged plans are a material help in the actual despatch of all Red Cross aid. In the present instance, however, exceptional difficulties have presented themselves. Some of these are due to the seat of war, for not only is the area of hostilities scattered and extensive, but the campaign may be largely waged in a mountainous and barren country, with few roads and under severe climatic conditions. Other hindrances arise from the fact that the belligerents include a number of different nationalities, whose armies, although armed with modern fire-arms, are not only unprovided with adequate and up-to-date medical arrangements, but have sanitary arrangements of a very primitive kind. From this it must follow that the sick and wounded are bound to be numerous, and that the facilities for meeting their needs and furnishing them with proper aid and treatment are likely to be limited.

In view of its decision to furnish aid to the sick and wounded of all the belligerents in the present war, the first step taken by the British Red Cross Society was to ascertain if it would be agreeable to all the combatants to receive it. Having learnt that the Governments of Bulgaria, Greece, Montenegro, Servia, and Turkey accepted with gratitude the offer of the Society to raise funds in order to send assistance to them, the sanction of our Foreign Office was then obtained for the despatch to the above countries of the proposed aid. Very full consideration was given to the question of the form it should take, and as it was strongly felt that the chief need at present was of surgeons, dressers, and male nurses, the decision come to was to organise and send out in the first instance complete male units furnished with separate medical and surgical equipment and with ample stores. Seeing that the centres of activity in the war area are at present likely to shift from place to place, we think that the plan adopted is the best one for the conditions that are likely to be met with. Such units being self contained and independent are capable of taking duty at the base or in the field, in the latter case keeping in touch with the moving front of the force and utilising any buildings met with as they go along.

### A Unit in Detail.

The personnel of such a Balkan Red Cross unit has been fixed at a total of eighteen, and is made up of three medical officers, three dressers (medical students), six nursing orderlies (one acting as sergeant-major), five general duty orderlies, and one cook. They are required to sign a contract for six months and undertake to wear Service uniform, which is supplied free, as are also ordinary boots, and, if specially ordered, india-rubber boots also. In addition, before embarking, each man is provided with brassard, haversack (fitted), mess tin (Cavalry), water bottle, clasp knife, sweater (or knitted waistcoat), three flannel shirts, three pairs socks, two pairs drawers, one blanket, one waterproof sheet (in which to roll the previous articles), and a first field dressing. The rates of pay are £1 a day for the medical officers, £2 per week for the dressers and for the sergeant-major, 30s. per week for nursing orderlies and for the cook, and 25s. a week for the general duty orderlies. If rations cannot be given, 5s. a day will be allowed in lieu of them. Medical officers are supplied with a copy of Spencer's "Military Surgery," of the R.A.M.C. Training Manual, and of the Scheme for the Organisation of Voluntary Aid in England and Wales. Candidates are selected by their professional knowledge and their physique, and they are allowed to make a choice as to the belligerent they would prefer to serve with, but no man is eligible for service if he is a reservist.

The medical and general equipment supplied to each unit corresponds very closely to that of the section of a field ambulance and consists of the following:—One pair field medical panniers, one pair field surgical panniers, one field medical companion, three pairs surgical haversacks, one field fracture box, one reserve dressing box, one medical comfort pannier, one set canteens A to H, one filter, four Red Cross flags, four Union flags, two camp kettles with contents, four stretchers, fifty brassards, one package sulphite of soda tabloids for water bottles, and fifty blankets. Extra stores can, of course, be sent in addition, but the above are considered ample for the unit and not too bulky to interfere with its mobility in the field.

Up to the present the British Red Cross Society has despatched six units to the seat of war, one to Montenegro, two to Greece, and three to Turkey. Help is, however, much wanted at Belgrade in Servia and at Sofia in Bulgaria, and it is hoped that it will be possible to send units to these countries as well. Although these Balkan Red Cross units are despatched for purely professional work, they have also a business side that has to be attended to, not the least important matter being their maintenance, a matter involving the securing of provisions, the payment of accounts, and the negotiating with the authorities. The care and distribution, too, of stores sent out has also to be provided for, and many other necessary details. In view of all this special work, the Society has appointed three directors to supervise these matters of administration, one of them doing duty in the Balkan States, another in Greece, and the third one in Turkey. This seems to us a very wise and proper step to have taken, and one that should increase the efficiency of the units.

As regards the cost of equipping and despatching a unit, it is estimated to range from £1,250 to £1,500, while its maintenance and the payment of its members for a period of six months may be calculated at not less than £4,000, but such a sum must be more or less problematical, for the cost of provisions and the amount of assistance that may be required from each unit cannot be accurately laid down. One thing only is sure, and that is that the amount of suffering and distress that will have to be dealt with is bound to be great and may surpass in its intensity anything experienced in modern wars. In view of this, it is hoped that there will be a generous response to the Balkan Fund, so that all the units sent out may be maintained. One thing the public may rest assured of, and that is, that the arrangements made by the British Red Cross Society are the best for meeting present needs and that everything has been done to see that the public money is being judiciously and wisely expended, seeing that the Society will be kept informed as to what are the most pressing necessities of the situation through their different directors.

